# Move Toward Recovery: A Feasibility Study for a Physical Activity Intervention to Reduce Post-Surgical Pain in Young Hispanic Breast Cancer Patients

**DOI:** 10.3390/ijerph23081003

**Published:** 2026-07-31

**Authors:** Stephanie Rosenberg, Tawny Boyce, Scott T. Walters, Laura Barriga, John Torres, Vernon Shane Pankratz, Bernard Tawfik, Sangeetha Prabhakaran, Stephanie Fine, Acadia W. Buro, Ursa Brown Glaberman, Cindy Blair, Jacklyn Nemunaitis

**Affiliations:** 1 Internal Medicine Department, University of New Mexico, Albuquerque, NM 87131, USA; vpankratz@salud.unm.edu (V.S.P.); btawfik@salud.unm.edu (B.T.); ciblair@salud.unm.edu (C.B.); jnemunaitis@salud.unm.edu (J.N.); 2 Comprehensive Cancer Center, University of New Mexico, Albuquerque, NM 87102, USA; tboyce@salud.unm.edu (T.B.); aburo@salud.unm.edu (A.W.B.); 3 Health Science Center, University of North Texas, Fort Worth, TX 76107, USA; scott.walters@unthsc.edu; 4 College of Education and Human Sciences, University of New Mexico, Albuquerque, NM 87131, USA; lbarriga@salud.unm.edu; 5 Health Sciences Center, University of New Mexico, Albuquerque, NM 87131, USA; johtorres@salud.unm.edu; 6 Department of Surgery, University of New Mexico, Albuquerque, NM 87106, USA; sprabhakaran@salud.unm.edu; 7 Christus St. Vincent Regional Cancer Center, Santa Fe, NM 87505, USA; stephanie.fine@christushealth.org (S.F.); ursa.brownglaberman@christushealth.org (U.B.G.); 8 College of Population Health, University of New Mexico, Albuquerque, NM 87131, USA

**Keywords:** breast neoplasms, mastectomy, cancer-related pain, pain management, post mastectomy pain syndrome, survivorship

## Abstract

**Highlights:**

**Public health relevance—How does this work relate to a public health issue?**

- Post-breast surgery pain syndrome (PBSPS) is one of the most common complications in young breast cancer survivors; it can impair physical activity and quality of life.
- Few exercise interventions have been designed with young Hispanic breast cancer survivors as the primary focus group.

**Public health significance—Why is this work of significance to public health?**

- A home-based physical activity and health coaching intervention using activity trackers and Spanish language delivery was found to be feasible, meeting recruitment and adherence rates, and retention approaching the prespecified target.
- Most participants remained engaged throughout the intervention and satisfaction was high.

**Public health implications—What are the key implications or messages for practitioners, policy makers and/or researchers in public health?**

- This work supports the feasibility of providing a flexible and remotely delivered exercise intervention for young Hispanic breast cancer survivors with post-breast surgery pain syndrome, while accommodating their everyday lives
- These findings support the use of flexible, technology-assisted physical activity programs as a scalable model for supportive care in cancer survivorship.

**Abstract:**

Young breast cancer survivors often develop post-breast surgery pain syndrome (PBSPS). Despite potential benefit from physical activity, access to in-person programs is often limited by competing work and caregiving responsibilities. We assessed the feasibility and acceptability of a remotely delivered physical activity intervention for young Hispanic breast cancer survivors with PBSPS. Our single-arm, 12-week feasibility study enrolled Hispanic women aged ≤60, at least 3 months post-treatment for invasive breast cancer or ductal carcinoma in situ (DCIS), with PBSPS. Using a Fitbit Inspire 3 activity tracker and health coaching, the program encouraged whole-of-day movement to increase activity. Feasibility outcomes included recruitment, retention, and adherence. Secondary outcomes assessed pain measures, daily steps, and quality of life (QOL). Twenty-five breast cancer survivors enrolled (mean age 47.4 years [SD 6.3]), with 76% selecting Spanish-language health coaching. Retention at 12 weeks was 79% (19/24). Engagement with health coaching was high, with 95% completing at least 4 of 5 pre-planned calls. The mean change in daily step count was 1502 (−348, 3353), with twelve (66.7%) increasing in step count (median 2334.5, range: 14, 13,422). Each additional 1000-step increase was associated with a 2.67-point increase in pain self-efficacy scores (β = 2.669, 95% CI: 0.884 to 4.454, *p* = 0.006). A remotely delivered, health coaching physical activity intervention was feasible and acceptable for young Hispanic breast cancer survivors with PBSPS. This supports further evaluation in a larger trial. Accessible survivorship interventions for young breast cancer patients in their preferred language are needed.

## 1. Introduction

Breast cancer is the most diagnosed cancer among women in the United States and the leading cause of cancer-related death among Hispanic women [1,2]. While breast cancer is more prevalent in older adults, younger women under 50 often experience more aggressive disease and undergo more intensive treatments [3]. One of the most common and burdensome complications in this population is persistent post-breast surgery pain syndrome (PBSPS), affecting an estimated 20–65% of patients [4]. The definition of PBSPS varies, but one widely accepted definition is pain in the ipsilateral breast/chest wall, axilla, or arm of the index breast surgery of at least moderate intensity (≥4 on a scale of 1–10), lasting at least 6 months, and occurring at least 50% of the time [5]. Among working women with PBSPS, 54% reported reducing workload to part-time as a direct result of the PBSPS pain [6]. This chronic pain can last for months or even years, impairing physical functioning, emotional wellbeing, and overall quality of life [4]. Existing pain management strategies include pharmacologic treatments and physical therapy, but these approaches are often insufficient, especially for underserved populations who may be limited by transportation and time constraints [7]. A scalable remote physical activity program may be better positioned to reach patients who cannot reliably attend in-person therapy. Evidence increasingly supports the role of physical activity in alleviating pain and improving other patient-reported outcomes, including anxiety, depression, and distress [8]. Additionally, recent studies of wearable technology-assisted interventions in breast cancer survivors found improvements in moderate-to-vigorous physical activity, total physical activity, and weight control [9]. Additionally, time limitations are a known barrier to participation in physical activity for young patients [10]. Virtual and flexible interventions may facilitate participation and adherence, and wearable activity trackers have shown promise for increasing physical activity among breast cancer survivors [11,12].

Despite this growing evidence, few exercise interventions have been designed with Hispanic breast cancer survivors as the primary focus group [13]. Although several studies have included substantial numbers of Hispanic participants, most were not tailored to Hispanic survivors nor were they focused on younger patients and pain-related outcomes such as PBSPS [13]. Minority status, including Hispanic ethnicity, has been identified as a risk factor for PBSPS. One possible explanation is that Hispanic patients are more likely to be diagnosed with later-stage disease, often requiring more extensive surgery [14]. Later-stage disease is also more likely to require additional neoadjuvant or adjuvant treatment (i.e., radiation or chemotherapy), which is also associated with high rates of pain [4]. Though national estimates do not consistently find lower mammogram use among Hispanic women [15], it’s been shown that Hispanic women experience delays in diagnostic evaluation following abnormal screening results [16]. This could be a contributing factor to more advanced disease at diagnosis. Younger patients are also at higher risk for developing PBSPS (Odds ratio [95% CI] of 1.36 [1.24–1.48] for every 10-yr decrement of age), though the cause for this is not clear, but may reflect a higher rate of adjunctive treatments or higher stage in this group [3]. These women often face unique recovery challenges due to the intensity of treatment, sociocultural factors, and limited access to support services [17]. Many young patients are also diagnosed amid forming relationships, raising families, and establishing a career and work–life balance, all of which are dramatically interrupted by their treatment and the consequences of that treatment. There is a need for accessible and flexible interventions tailored to the needs of this younger population.

To address this gap, we evaluated the feasibility of a 12-week, home-based physical activity intervention for young Hispanic women with PBSPS. The intervention included Fitbit activity trackers for self-monitoring and goal setting, along with individually tailored sessions with health coaches trained in motivational interviewing. The intervention used a whole-of-day approach to physical activity, which focuses on the accumulation of intermittent bouts of activity (stepping) throughout the day [18]. The intervention is remotely delivered and individually tailored, thus lowering barriers to participation such as travel and time constraints. The study’s primary focus was to evaluate the intervention’s feasibility, safety, and acceptability for patients, and secondarily assessed effects on pain, physical activity, and health-related quality of life (hrQOL). To our knowledge, there are no studies of this kind focusing on improving PBSPS outcomes in young, Hispanic breast cancer survivors. Ultimately, this work aims to improve long-term outcomes for young, Hispanic women recovering from curative-intent breast cancer treatment.

## 2. Materials and Methods

### 2.1. Study Setting

The Move Toward Recovery study was conducted at the University of New Mexico (UNM) Comprehensive Cancer Center in Albuquerque, New Mexico, which serves a large and diverse Hispanic community. Approximately 55% of the patients served at UNM Hospital are Hispanic. The study employed a remotely delivered intervention within a single-arm pre–post design. This study received approval from the Institutional Review Board (IRB) at the University of New Mexico Health Sciences Center. Informed consent was obtained from all participants prior to their involvement in the study.

### 2.2. Participants

The accrual goal was 25 breast cancer patients. Providers identified patients who met the study definition of post-breast surgery pain syndrome (PBSPS), defined as pain in the area of breast surgery, located in the ipsilateral breast/chest wall, axilla, or arm, lasting at least 6 months, and occurring at least 50% of the time [5]. Additional eligibility criteria included: (1) completion of primary treatment for invasive breast cancer or ductal carcinoma in situ (DCIS) at least 3 months before enrollment, and having undergone any breast surgery (lumpectomy or mastectomy); (2) aged 60 years or younger; (3) self-identified as Hispanic. Ongoing endocrine therapy and ovarian suppression were allowed. The study excluded patients with metastatic or locally recurrent disease with no curative intent treatment, those who met or exceeded physical activity guidelines (>150 min/week of moderate-intensity exercise), those unable to speak, read, or understand English or Spanish, those experiencing severe impairments in seeing or hearing or pre-existing medical limitations for engaging in daily physical activity, or those unwilling to use their own or a study provided cell phone and/or wear activity monitors.

### 2.3. Intervention

The intervention was guided by self-determination theory and social cognitive theory and used motivational interviewing to promote behavior change. Details of the conceptual framework, goals, and components of this intervention were published previously [18]. Using a whole-of-day approach, the first of the secondary goals was to increase participants’ activity levels throughout the day without emphasizing the intensity or duration of individual activity bouts. The overall step goal was to increase an additional 3000 steps above baseline. We encouraged incremental and achievable step goals to increase self-efficacy. For example, the step goal for the first two weeks was to increase an additional 1000 steps/day above baseline. The second goal was to achieve at least 10 active hours per day (defined as >250 steps/hour by activity device).

Participants were mailed a Fitbit Inspire 3 tracker and an instruction booklet on how to set up the device and how to download the free phone app, along with visual aids for the health coaching sessions. Health coaching calls were conducted in Spanish or English depending on patient preference and occurred every other week. Health coaches, trained in motivational interviewing, guided participants in setting up the devices to collect baseline data during week 2. Starting in week 3, they facilitated discussions to elicit change talk, improve self-efficacy, enhance motivation, and support autonomy to select strategies to increase physical activity. The educational materials (visual aids) used during the health coaching sessions included: (a) the benefits of physical activity and suggestions for how to add more steps to their day, throughout the day (i.e., breaking up sedentary time through at least minimal stepping each hour); (b) reasons to be physically active; (c) example plans for meeting step goals; (d) ideas for getting more steps; and (e) pages for the participant to write their plan for each week.

### 2.4. Data Collection

Upon enrollment, participants received a baseline assessment package, which included a survey packet, an activPAL monitor, and instructions for completing the assessment. The equipment was sourced from PAL Technologies Ltd, Glasgow, United Kingdom. The same assessment was repeated at the end of the intervention (week 13). The activPAL monitor tracked standing, sedentary time, and steps [19,20]. Participants were instructed to apply and wear the activPAL activity monitors for 1 week, ideally for 24 h/day, i.e., a 24 h protocol, consistent with other studies [21,22,23]. The intervention was delivered over 12 weeks, with a post-intervention assessment in week 13. Participants had the option of completing the surveys on paper or online, via REDCap version 16.0.40. Study data were collected and managed using REDCap electronic data capture tools hosted at the University of New Mexico [24,25].

### 2.5. Outcomes and Measures

The primary aim of this study was to assess feasibility by recruiting 25 breast cancer survivors within 10 months, achieving 80% retention (completion of all pre- and post-assessments) and 80% adherence (completing at least 4 of 5 health coaching sessions), while minimizing adverse events. These goals were based on our power analysis and common target of 80% in feasibility studies [26]. Acceptability and satisfaction with the program were assessed via a 14-item survey (6-item Likert scale, “strongly disagree” to “strongly agree”, plus not applicable).

Secondary outcomes included pain-related measures, daily steps, and QOL. Pain was assessed using the Defense and Veterans Pain Rating Scale, which is an easy-to-use, reproducible visual scale, validated in cancer patients [27]. Pain interference was assessed using a 1–10 numeric rating scale across four domains: usual activity, sleep, mood, and stress. Scores ranged from zero (“does not interfere”) to 10 (“completely interferes”). Other pain-related measures included the Bodily Threat Monitoring Scale (BTMS) and the pain self-efficacy questionnaire (PSEQ) [28,29]. The BTMS assessed attentional focus and worry related to bodily sensations (19 questions). Response items are on a 5-point Likert scale (“not at all like me” to “entirely like me”). Total scores were sums of the 19 items with a range from 0 to 76, with higher scores reflecting greater vigilance and concern. The Bodily Monitoring Subscale was a sum of 6 items resulting in scores ranging from 0 to 24, and Bodily Threat Appraisals Subscale scores were a sum of the remaining 13 items with a range from 0 to 52. The Pain Self-Efficacy Questionnaire (PSEQ) includes 10 questions to assess confidence despite having pain. Responses range from “not at all confident” (score = 0) to “completely confident” (score = 6). PSEQ domains include areas such as household chores, socialization, coping with pain, work, hobbies, leisure activity, accomplishing life goals, and living a normal lifestyle. Overall PSEQ score was a sum of the 10-items, resulting in a score ranging from 0 to 60, where scores 0–29 reflect “low self-efficacy, 30–40 reflect “moderate self-efficacy”, and scores 40–60 reflect “high self-efficacy”. QOL was measured using PROMIS (Patient-Reported Outcomes Measurement Information System) measures [30]. The 8-item short forms assess domains in mental health (anxiety and depression), physical health (physical function, fatigue, sleep disturbance, and sleep impairment), and social health (satisfaction with social roles and activities, i.e., social functioning). These instruments are valid and reliable for use in diverse clinical samples [31,32,33,34]. Surveys were scored using the free Health Measures Scoring Service [35]. The service provides T-scores, which represent a linear transformation of the raw scores, normed to the general population, with a mean of 50 and a standard deviation of 10. Higher scores represent greater symptom burden for anxiety, depression, fatigue, and sleep disturbance, and greater functioning for social participation and physical function. Sociodemographic, health-related characteristics, and medical history were collected and used to characterize the study population and/or as predictors of adherence.

### 2.6. Statistical Analysis

Descriptive statistics were used to summarize participant demographics, intervention adherence, and retention rates. These were reported as counts and percentages for categorical variables and means and standard deviations (SD) for continuous values. Primary analyses focused on evaluating recruitment (25 participants within 10 months), retention (completion of all pre- and post- assessments), and adherence (completion of at least four of five health coaching sessions) as percentages of enrolled participants. This recruitment goal was based on the common target of 80% enrollment in feasibility studies; enrolling 25 participants would enable us to estimate retention rates to within plus or minus 16% if expected retention is 80. Also, with 25 participants and at least 80% retention, we would be able to estimate the mean change scores to within plus or minus one-half of the standard deviation of the change scores [36]. Secondary objectives assessed the impact of the intervention on pain scores, physical activity, and hrQOL. Given the exploratory nature of this feasibility study, analyses of these secondary outcomes were considered hypothesis-generating. Physical activity measures were based on activPAL data for wear times greater than 12 h/day for the week of pre- and post-assessment. Median values were calculated to describe daily step count, sedentary time, and standing time for each participant.

To assess the potential impact of the intervention on these measures, we computed the change scores for each of the measures as the difference between the post- and pre-assessments (post minus pre). We summarized these change scores with means and standard deviations and estimated 95% confidence intervals and performed paired *t*-tests to assess the significance of these within-person changes over time. Additionally, for each of the pain scales and PROMIS measures, simple linear regression was used to estimate the association between change in step count (per 1000 steps) and the change in pain scale measures and report these regression coefficient (β) estimates and their 95% confidence intervals. We calculated paired-sample Cohen’s *d* effect sizes for all pre–post comparisons to complement other estimates and facilitate interpretation of the magnitude of change. If Cohen’s *d* is <0.20, then magnitude is “trivial”; for 0.20–0.50, then magnitude is “small”; for 0.50–0.80, then magnitude is “medium”; for >0.80, then magnitude is “large”. Pearson correlation coefficients were obtained as additional summaries of the strength of these associations modeled via linear regression. These are univariate regression analyses given the small sample size. A nominal type 1 error level of 0.05 was applied to assess the potential significance of the statistical comparisons made for these secondary analyses. Statistical analyses were conducted using SAS 9.4 software.

## 3. Results

### 3.1. Patient Demographic and Clinical Characteristics

**Figure 1:**
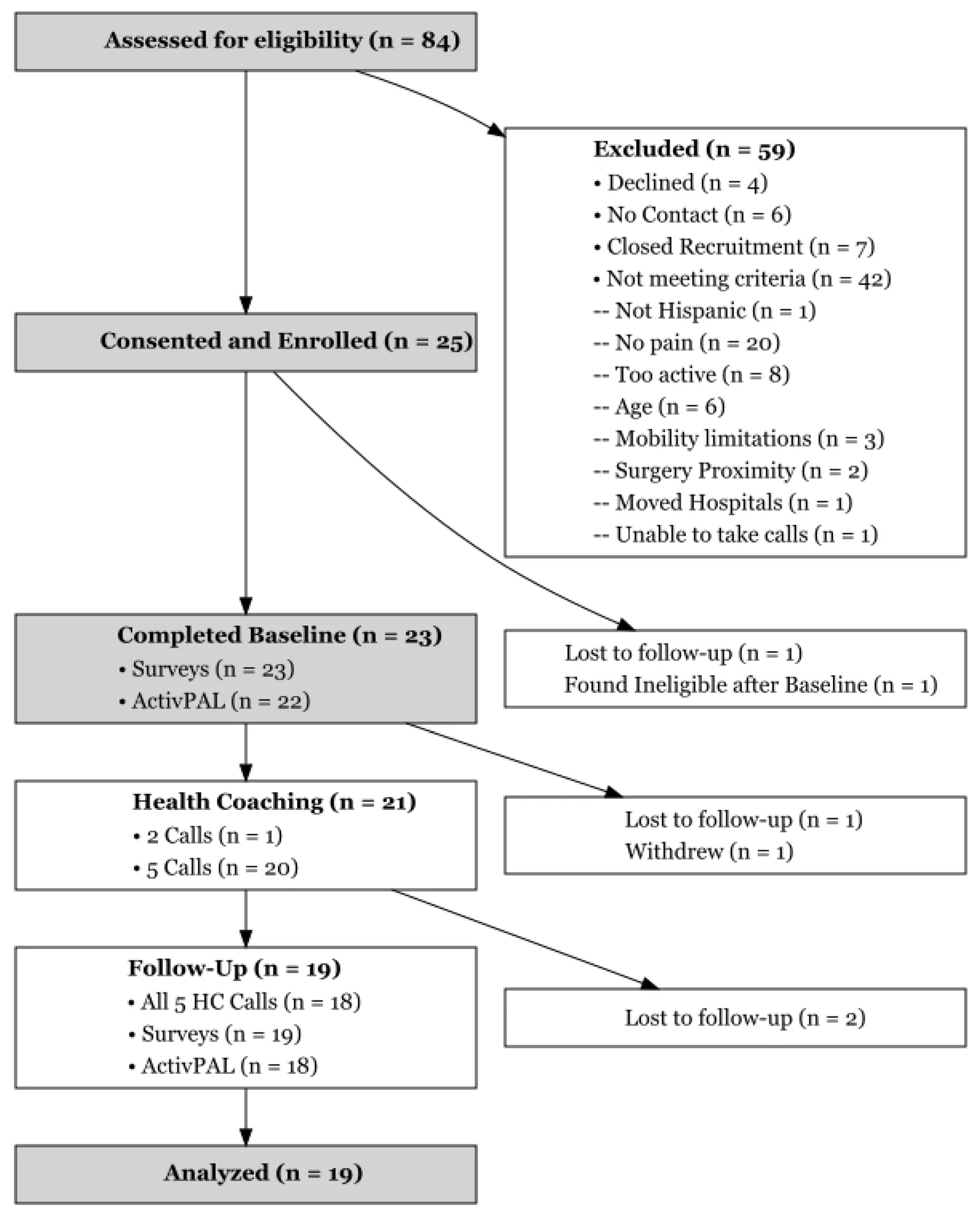
Consolidated standard of reporting trials (CONSORT) diagram.

Overall study feasibility was assessed through recruitment, retention, and intervention adherence, as summarized in the CONSORT (Figure 1). Of the 84 individuals screened within the nine-month enrollment period (December 2023–August 2024), 59 were deemed ineligible primarily due to absence of pain, high baseline activity levels, or age criteria. A total of 25 were consented and enrolled (recruitment rate of 59.5%). One participant was found to be ineligible after enrollment, and one participant was lost to follow-up. Twenty-three participants completed baseline assessments, including surveys and objectively measured physical activity (steps). Overall, nineteen participants completed follow-up assessments and were included in the final analysis, representing a 79% retention rate among eligible enrollees. Reasons for dropout included discomfort with technology (*n* = 1), diagnosis of a second cancer (*n* = 1), and loss to follow-up (*n* = 3) after no response to contact attempts. Adherence to the intervention was high among the 21 who participated in health coaching, with 20 completing all five coaching sessions. Completion of follow-up assessments was similar, with surveys completed by all 19 retained participants and activity monitoring completed by 18 of 19 participants. Baseline participant data was collected from January 2024 through September 2024, and follow-up data was collected from April 2024 through January 2025.

Participants had a mean age of 47.4 years, and all identified as Hispanic (Table 1). The study sample was socioeconomically diverse, with variation in education and household income. The largest proportion had completed high school or a GED as the highest education level, and nearly half reported annual household incomes below $30,000. Spanish was the primary language of the participants (65.2%), with 76.0% selecting Spanish for the study intervention. Household structures varied, with many participants living with a spouse or partner (39.3%) and children (47.8%). Most supported multiple dependents, commonly three to four individuals.

Baseline clinical characteristics are summarized in Table 2. About half were diagnosed with early-stage disease (stage I–II; 56.5%), and the other half late stage (stage III; 43.5%). The majority underwent mastectomy (71.4%), received adjuvant therapy (56.5%) and radiation (78.3%), with a mean time since surgery of 3.78 years (SD 2.89). Most were referred to physical therapy for pain (65.2%), and (52.2%) felt that they were in good health.

Acceptability of the intervention is described in Table 3, with percentages of agreed and strongly agreed. There was high acceptability of the Fitbit tracker and phone app, including ease of use and motivation (82% to 100%). For health coaching, there was also high acceptability. All participants (100%) reported that they enjoyed talking with the health coach, and 88.2% reported that the health coaching helped them identify strategies. Satisfaction with the intervention was very high, with 94% willing to continue using the Fitbit and 100% recommending the program to other breast cancer survivors.

Feasibility of activPAL wear for pre and post was assessed by the number of valid wear days completed at each assessment timepoint. Among the 19 participants, 18 wore the monitor for both assessments. The median number of wear days was 6.5 days (range: 4–7) at the pre-assessment and 6.0 days (range: 3–7) at the post-assessment, indicating generally high adherence to activPAL wear throughout the study period.

### 3.2. Secondary Outcomes Related to Pain

Participants reported numerical decreases in pain interference across all domains (Table 4), although only the change in pain interference with stress was statistically significant. For this outcome, the proportion of individuals reporting no interference with stress increased from 8% to 21%, and the percentage of participants endorsing complete interference with stress decreased (from 8.7% to 5.3%). Mean scores for the “pain interfering with stress” outcome decreased by 1.7 points (95% CI for mean decrease ranged from 0.19 to 3.17).

The mean BTMS scores decreased numerically from 44.84 at baseline to 36.37 at follow-up, suggesting a potential reduction in threat monitoring behaviors (mean change, 95% CI: −8.47, −17.3 to −0.3, Table 5). The change in the BTMS score was driven primarily by the bodily threat appraisals subscale, with questions regarding bodily sensations. Participants worried less about different bodily sensations (mean change, 95% CI: −0.79, −1.46 to −0.12) or new bodily sensations in case they meant something was wrong (mean change, 95% CI: −0.74, −1.36 to −0.12). Additionally, their enjoyment was numerically less affected by worry that something is wrong with their body (mean change, 95% CI: −0.79, −1.60 to 0.02). However, these changes did not meet the pre-defined threshold and are therefore not statistically significant.

Participants demonstrated numerical improvements in each item of the PSEQ following the intervention. These included household chores, socializing, coping with pain, work, hobbies, leisurely activity, and accomplishing life goals. A numerical increase was seen in participant confidence in gradually becoming more active despite pain (0.89, CI 0.11–1.68; *p* = 0.028). Although the total PSEQ score (self-efficacy to manage pain) increased (4.89, CI −2.69 to 12.48; *p* = 0.192), this was not statistically significant, suggesting that the item-specific summaries should also be considered to reflect exploratory assessments only.

### 3.3. Secondary Outcome of QOL

Participant QOL scores by domain are presented in Table 6. At study baseline, QOL scores ranged from 0.5–1.0 SDs worse than the general population for all domains except social participation. On average, anxiety, depression, fatigue, and sleep disturbance each demonstrated statistical significance in their improvements of 4 to 5 points (Cohen’s d values ≈ 0.5). Domains of physical function, social participation, and sleep-related impairment were not significantly improved, although numerical improvements of ≈3 points (Cohen’s d values < 0.5) were observed.

### 3.4. Secondary Outcome of Physical Activity (Steps)

At study baseline, the average daily step count shown in Table 7 was relatively high (mean: 6737 steps/day). The mean change in daily step count was 1502 (−348, 3353). Change in step count was variable. Of the 18 participants with pre- and post-activPAL data, twelve (66.7%) participants showed an increase in step count (Median: 2334.5, Range: 14, 13,422), while six participants showed a decrease in steps (Median: −938.5, Range: −5156, −117). Changes in sedentary time and standing time were minimal.

Table 8 shows the association between change in daily step count and change in Pain Scales and QOL scores after the 12-week health coaching intervention. The regression coefficients (β) represent the expected change in each outcome for every 1000-step increase in step count. A 95% confidence interval is provided for each estimate.

For pain interference with activity, sleep, mood, and stress, each additional 1000-step increase was associated with small non-significant decreases in pain scores. Higher BTMS scores indicate a greater tendency to monitor bodily sensations and interpret them as threatening, and no significant association was found between change in step count and changes in any of the BTMS measures. A positive association was observed between increased step count and improvement in pain self-efficacy. Each additional 1000-step increase was associated with a 2.67-point increase in PSEQ scores (β = 2.669, 95% CI: 0.884 to 4.454, *p* = 0.006).

Increases in step count were also associated with improvements in several important patient-reported outcomes, particularly lower depression (β = −1.400, 95% CI: −2.435 to −0.366, *p* = 0.011), better social participation (β = 1.628, 95% CI: 0.703 to 2.553, *p* = 0.002), improved physical function (β = 0.927, 95% CI: 0.052 to 1.802, *p* = 0.039), and better sleep related outcomes (sleep disturbance: β = −1.444, 95% CI: −2.479 to −0.408, *p* = 0.009 and sleep-related impairment: β = −1.413, 95% CI: −2.340 to −0.485, *p* = 0.005). Participants who increased their steps tended to report better ability to participate in social roles and activities. Each additional 1000 steps was associated with a 1.63-point increase in social participation scores (β = 1.628, 95% CI: 0.703 to 2.553, *p* = 0.002). There was no statistically significant association between change in steps and change in anxiety or fatigue scores.

## 4. Discussion

This study demonstrated the feasibility of a remotely delivered physical activity intervention among young Hispanic women with persistent PBSPS, a group underrepresented in survivorship research. High engagement with health coaching and retention approaching the prespecified target support the acceptability of this approach. Of the 24 who were successfully eligible and enrolled, 21 fully engaged in the health coaching sessions. Socio-demographic characteristics were available from 19 participants, a cohort that represents survivors at varied disease stages, several years post-surgery with persistent symptoms despite being referred for supportive care. Their summaries reflect the environment that many younger Hispanic breast cancer patients navigate in recovery, where they balance family responsibilities, employment, and financial strain alongside symptom management. Additionally, chronic pain can contribute to lost productivity, disability, and increased healthcare utilization [37]. It is also linked to social isolation and reduced social participation, which can further worsen mental and physical health over time [38].

Our findings are relevant to our population of young Hispanic women, who are more likely to present with advanced disease, require intensive multimodal therapy, and lack access to supportive care programs. Current American Society of Clinical Oncology (ASCO) survivorship guidelines emphasize the role of exercise and structured activity as integral to recovery and long-term health in cancer survivors [13]. Gonzalo-Encabo et al. described a 12- to 16-week combined aerobic and resistance training program that reduced cardiovascular risk factors in Hispanic and Latina breast cancer patients, finding functional and cardiometabolic benefits in this population [39]. Others have shown that targeted exercise counseling was associated with improved exercise participation and quality of life in breast cancer survivors [40]. While several exercise interventions have included substantial numbers of Hispanic participants, the ASCO Educational Review notes that Hispanic breast cancer survivors have infrequently been the primary target of aerobic exercise trials, and that recruitment, retention, and adherence outcomes have varied across interventions [13]. Our study retained 79% of younger Hispanic breast cancer survivors in a home-based physical activity intervention. Although our retention fell slightly below the predetermined retention goal of 80%, the remaining goals of sustained engagement were achieved, supporting the overall feasibility of the intervention. It is important to consider that it’s easier to have high retention rates when enrolling survivors who are relatively healthy, well-educated, and have sufficient income. This study enrolled a very diverse sample of breast cancer survivors, contributing to the literature in reaching this population. After consent and enrollment, a total of 4 patients were lost to follow-up. This presents an opportunity in future study design to strengthen retention.

Recruitment and retention of participants in this study demonstrate the feasibility of delivering a technology-assisted, home-based physical activity program to this population. High levels of adherence to health coaching sessions and device use indicate that patients found the intervention acceptable. This is consistent with current evidence that wearable technology combined with individualized coaching can promote engagement in cancer survivors. A recent meta-analysis of wearable technology-assisted interventions in breast cancer survivors found improvements in moderate-to-vigorous physical activity, total physical activity, and weight control [9]. Scoping reviews similarly show that devices like Fitbit and accelerometers are acceptable, improve self-awareness, and produce short-term gains in activity, though there can be attenuation over time. Importantly, ASCO emphasizes accessible and equitable care as key domains and recommends integrating evidence-based physical activity and technology into patient care plans [41]. The flexible whole-of-day approach used in this study may have lowered barriers to participation compared to more structured exercise regimens, which can require dedicated time, travel, or facilities. This is relevant to our population, which often balances caregiving, work, language, and financial stressors. At the same time, there can be challenges with technology-based methods. Devices can suffer from wear-time nonadherence. Additionally, user engagement and motivation may decline over time. Data syncing, digital literacy, and technical support are all important considerations. Some studies note that increases in activity from wearable device interventions tend to decrease in the maintenance phase [42].

Persistent pain is clinically meaningful because it is associated with higher depressive symptoms, anxiety, and worry about recurrence [43]. In our participants, there was reported change across multiple patient-reported domains from baseline to follow-up. Pain interference scores improved in measures of stress, which could indicate that participants experienced less disruption from pain in their daily lives. Similarly, the BTMS showed less hypervigilance and worry toward bodily sensations, which may reflect improved confidence in physical function and less fear surrounding their symptoms. PROMIS measures similarly supported improvements in measures of daily life, particularly sleep disturbance and fatigue. Together, these findings suggest that the intervention may have multiple benefits beyond pain alone. This aligns with prior research using PROMIS and related measures in breast cancer survivors [44].

Additionally, our results support the conduct of a larger, controlled trial to evaluate the efficacy and benefits of our intervention. In addition to daily activity, future studies could explore composite measures, integrating daily pain and physical activity patterns. This could reflect more favorable days (high activity with low pain), and lower scores to reflect less favorable days (low activity with high pain). A compound variable reflects the added burden associated with maintaining activity despite pain symptoms, which could further assess the impact of health-coaching, activity monitor use, or other interventions to increase physical activity. Because participants already had relatively high baseline step counts, future studies could consider including work-related physical activity in screening for eligibility. We did not formally measure digital literacy during this study and therefore cannot determine if technology-related challenges contributed to retention. Future studies could assess participant digital literacy and technical support needs. Cost-effectiveness studies are needed to understand the value of lowering healthcare utilization with home-based activity programs. Lastly, studies with longer follow-up are needed to assess whether improvements persist after coaching support is withdrawn.

This feasibility study has several limitations that should be considered. Primarily, it was a single-center study with a small sample size, which limits generalizability. It was also a single-arm study that measured change over time for the secondary outcomes, making it necessary to limit interpretation of these relationships as correlative rather than causative. When considering the relationships identified in our secondary analyses, the results should only be interpreted as exploratory given the feasibility design, small sample size, and multiple outcomes evaluated. The enrolled patients largely reported the presence of pain, but with relatively low initial scores, which limited the study’s ability to demonstrate change in this outcome. The single-arm design limits the ability to differentiate the intervention effect from the natural progression of recovery post-surgery. For patients with PBSPS, pain and quality of life may improve over time for some, but a substantial portion report persistent pain beyond 6 to 12 months and even years, and the literature does not provide a definitive rate of pain resolution over time [45]. Attrition from baseline to follow-up (19 of 24 participants completing) could introduce bias if those who discontinued had higher symptom burden or less digital literacy. Outcomes were mainly self-reported, which can be subject to recall and response bias. Although wearable devices provide objective data, step counts may not have fully captured the quality or intensity of activity or non-stepping activity (e.g., swimming). Future studies could incorporate complementary objective activity assessments, such as the 6-min walk test, to provide a more comprehensive evaluation of physical function. The high baseline step counts in this group further complicates interpretation, as there are likely ceiling effects. Future studies could consider grouping participants by their starting activity levels before randomization. This study was conducted at a cancer center with resources for recruitment and bilingual support, which may not be available in community or rural settings. Lastly, outcomes were assessed only at the completion of the 12-week intervention. The study did not include a long-term follow-up assessment of steps, pain, bodily threat monitoring, and QOL domains, to see if improvements are sustained over time. Future studies should evaluate whether these improvements are sustained over longer periods of time. This study has several strengths. The use of bilingual health coaching with motivational interviewing, combined with wearable technology, supports accessibility and flexibility, and is culturally relevant. High adherence to coaching sessions and Fitbit use demonstrates feasibility and acceptability, even in participants with limited experience tracking their steps. Importantly, the study collected a comprehensive set of outcomes, including pain interference scores, bodily threat monitoring, and multiple QOL domains. While this feasibility study lacked a control group, our analyses showed associations between increased step counts and reduced depression, sleep disturbance, and sleep-related impairment. These design elements support the potential of this intervention as a practical model for survivorship care.

## 5. Conclusions

This study demonstrates the feasibility and acceptability of a home-based physical activity intervention that integrates wearable technology and health coaching for young Hispanic women with pain after breast cancer surgery. Recruitment and adherence rates were met, and retention approached the prespecified target. Participants engaged consistently with both coaching sessions and device use despite varying levels of digital literacy. Preliminary improvements in pain, fatigue, and psychosocial wellbeing suggest that this approach may enhance recovery while accommodating the everyday lives of our patients. These findings support the use of flexible, technology-assisted physical activity programs as a scalable model for supportive care in cancer survivorship.

## Figures and Tables

**Table 1:** Demographic characteristics of baseline participants (*n* = 23).

Characteristics	Value
Age, N, mean (SD)	23, 47.4 (6.3)
Ethnicity	
Hispanic	23 (100.0%)
Language Spoken at Home	
English	6 (26.1%)
Spanish	15 (65.2%)
English & Spanish Equally	2 (8.8%)
Household Structure	
Spouse, partner	9 (39.1%)
Children	11 (47.8%)
Other relatives	1 (4.3%)
Alone	2 (8.7%)
Education	
High school, GED, or less	12 (52.2%)
Some additional schooling	5 (21.7%)
College, graduate, professional degree	4 (17.4%)
No answer	2 (8.7%)
Employment	
Employed for wages	15 (65.2%)
Self-employed	4 (17.4%)
Unemployed	1 (4.4%)
Homemaker	3 (13.0%)
Household income	
Less than 30,000	11 (47.8%)
30,000–49,000	6 (26.1%)
Greater than 70,000	3 (13.0%)
No answer	3 (13.0%)
Individuals supported by income	
1–2	7 (30.4%)
3–4	12 (52.2%)
5–6	2 (8.7%)
No answer	2 (8.7%)

**Table 2:** Clinical characteristics of baseline participants (*n* = 23).

Characteristics	Value
Stage at Diagnosis	
Early stage	13 (56.5%)
Late stage	10 (43.5%)
Surgical Type	
Mastectomy	15 (71.4%)
Lumpectomy	6 (28.6%)
Sentinel lymph node biopsy	7 (46.7%)
Axillary lymph node dissection	8 (53.3%)
Was patient referred to physical or occupational therapy?	
Yes	15 (65.2%)
No	7 (30.4%)
No answer	1 (4.3%)
Adjuvant Therapy	
Yes	13 (56.5%)
No	10 (43.5%)
Radiation	
Yes	18 (78.3%)
No	5 (21.7%)
Years since surgery	
Mean	3.78 (SD 2.89)
Smoking status	
Not currently smoking, never smoker	13 (56.5%)
Currently smoking, past smoker	10 (43.5%)
Self-assessment of health	
Good	12 (52.2%)
Fair	7 (30.4%)
Poor	4 (17.4%)

**Table 3:** Acceptability of intervention using 14-item survey (6-item Likert scale, strongly disagree to strongly agree, plus not applicable) in participants who completed the intervention (*n* = 19).

Survey Item	Percentage of Agree and Strongly Agree
Will recommend program to others	100%
Will recommend Fitbit to others	100%
Enjoyed talking with the health coach	100%
Motivated me to get more steps	100%
Will continue to use Fitbit	94.10%
Typically checked steps on mobile app	94.10%
Helped me meet step goals	94.10%
Tracker was easy to use	94.10%
More aware of activity	94.10%
Would do the study again	88.20%
Tried to get more steps with tracker vibration	88.20%
Mobile app was easy to use	88.20%
Health coach helped me identify strategies	88.20%
Reminders to move were helpful	82.40%

**Table 4:** Pain interference outcomes pre- and post-intervention.

Outcome	N	Pre-Mean (95% CI)	Post-Mean (95% CI)	Change Mean (95% CI)	Cohen’s *d*	*p*-Value
Pain interfering with your usual activity	18	3.72 (2.59, 4.85)	2.89 (1.38, 4.39)	−0.83 (−2.31, 0.65)	−0.28	0.25
Pain interfering with sleep	18	3.72 (2.21, 5.24)	2.44 (0.86, 4.03)	−1.28 (−3.07, 0.51)	−0.36	0.15
Pain interfering with mood	18	3.61 (2.49, 4.73)	2.89 (1.51, 4.26)	−0.72 (−1.96, 0.52)	−0.29	0.236
Pain interfering with stress	19	4.53 (3.01, 6.05)	2.84 (1.48, 4.21)	−1.68 (−3.17, −0.19)	−0.54	0.029

Values are mean (95% confidence interval), *p*-values from paired *t*-tests of change pre to post.

**Table 5:** Bodily threat monitoring score (BTMS) outcomes pre- and post-intervention.

BTMS Item	N	Pre-Mean (95% CI)	Post-Mean (95% CI)	Change Mean (95% CI)	Cohen’s *d*	*p*-Value
Bodily Threat Monitoring Scale (0–76)	19	44.84 (34.82, 54.87)	36.37 (26.05, 46.69)	−8.47 (−17.3, 0.31)	−0.47	0.058
Bodily Monitoring Subscale (0–24)	19	15.32 (12.41, 18.22)	12.95 (9.66, 16.23)	−2.37 (−5.37, 0.63)	−0.38	0.114
Bodily Threat Appraisals Subscale (0–52)	19	29.53 (22.06, 36.99)	23.42 (16.15, 30.70)	−6.11 (−12.5, 0.26)	−0.46	0.059

Higher scores indicate a greater tendency to monitor for and interpret bodily sensations as signs of something being wrong.

**Table 6:** PROMIS outcomes at pre- and post-intervention.

PROMIS Measure	*N*	Pre-Mean (95% CI)	Post-Mean (95% CI)	Change Mean (95% CI)	Cohen’s *d*	*p*-Value
Anxiety	19	60.21 (54.57, 65.84)	56.24 (51.36, 61.12)	−3.97 (−7.57, −0.37)	−0.53	0.033
Depression	18	54.44 (48.62, 60.27)	49.97 (44.65, 55.28)	−4.48 (−8.42, −0.54)	−0.57	0.028
Fatigue	19	57.11 (52.20, 62.01)	51.92 (47.41, 56.43)	−5.19 (−9.96, −0.41)	−0.52	0.035
Social Participation	19	48.79 (44.58, 53.01)	52.18 (47.97, 56.40)	3.39 (−0.91, 7.69)	0.38	0.115
Physical Function	19	44.45 (41.02, 47.88)	47.68 (43.98, 51.38)	3.23 (−0.09, 6.55)	0.47	0.056
Sleep Disturbance	19	58.47 (54.98, 61.97)	53.11 (48.69, 57.52)	−5.37 (−10.1, −0.69)	−0.55	0.027
Sleep-Related Impairment	19	57.28 (53.94, 60.63)	54.57 (50.06, 59.08)	−2.72 (−6.78, 1.35)	−0.32	0.178

Higher scores indicate more of the outcome.

**Table 7:** Change in activPAL physical activity measures pre vs. post.

Measure	*N*	Pre-Mean (95% CI)	Post-Mean (95% CI)	Change Mean (95% CI)	Cohen’s *d*	*p*-Value
Step Count (per day)	18	6737.0 (5435.9, 8038.1)	8239.3 (6276.8, 10,201.8)	1502.3 (−348.0, 3352.5)	0.40	0.105
Total Sedentary Time (min/day)	18	525.4 (468.0, 582.7)	501.5 (423.3, 579.6)	−23.9 (−84.3, 36.5)	−0.20	0.415
Standing Time (min/day)	18	315.5 (254.3, 376.8)	319.9 (253.6, 386.3)	4.4 (−40.8, 49.6)	0.05	0.840

Paired *t*-test *p*-value.

**Table 8:** Association between change in step count (per 1000) and change in pain and health-related quality of life outcomes.

Outcome	β per 1000 Steps (95% CI)	SE	r	*p*-Value
Pain Interference				
Pain interfering with your usual activity	−0.023 (−0.468, 0.421)	0.208	−0.029	0.912
Pain interfering with sleep	−0.200 (−0.728, 0.328)	0.248	−0.204	0.432
Pain interfering with mood	−0.079 (−0.447, 0.290)	0.173	−0.117	0.655
Pain interfering with stress	−0.031 (−0.481, 0.420)	0.213	−0.036	0.887
Bodily Threat Monitoring Scale 0–76 *	0.218 (−2.348, 2.785)	1.211	0.045	0.859
Bodily Threat Appraisals Subscale 0–52 *	0.255 (−1.593, 2.104)	0.872	0.073	0.773
Bodily Monitoring Subscale 0–24 *	−0.037 (−0.934, 0.860)	0.423	−0.022	0.931
Pain Self Efficacy Scale Score (/60) †	2.669 (0.884, 4.454)	0.842	0.621	0.006
Quality of Life				
PROMIS Anxiety ‡	−0.342 (−1.408, 0.723)	0.503	−0.168	0.506
PROMIS Depression ‡	−1.400 (−2.435, −0.366)	0.485	−0.597	0.011
PROMIS Fatigue ‡	−0.772 (−2.036, 0.493)	0.596	−0.308	0.214
PROMIS Ability to Participate Socially ‡	1.628 (0.703, 2.553)	0.436	0.682	0.002
PROMIS Physical Function ‡	0.927 (0.052, 1.802)	0.413	0.490	0.039
PROMIS Sleep Disturbances ‡	−1.444 (−2.479, −0.408)	0.488	−0.594	0.009
PROMIS Sleep-Related Impairment ‡	−1.43 (−2.340, −0.485)	0.438	−0.628	0.005

* Higher scores indicate a greater tendency to monitor for and interpret bodily sensations as signs of something wrong. † Higher scores indicate greater self-efficacy for managing pain. A score of 60/60 represents 100% confidence. ‡ Higher scores indicate more of the outcome.

## Data Availability

The data that support the findings of this study are available from the corresponding author upon reasonable request.

## Abbreviations

The following abbreviations are used in this manuscript:

PBSPS	Post-breast surgery pain syndrome
hrQOL	Health-related quality of life
DCIS	Ductal carcinoma in situ
IRB	Institutional review board
BTMS	Bodily threat monitoring scale
PROMIS	Patient reported outcomes measurement information system
ASCO	American society of clinical oncology
UNM	University of New Mexico
